# Integrated Transcriptomic and Metabolomic Analysis of Five *Panax ginseng* Cultivars Reveals the Dynamics of Ginsenoside Biosynthesis

**DOI:** 10.3389/fpls.2017.01048

**Published:** 2017-06-19

**Authors:** Yun Sun Lee, Hyun-Seung Park, Dong-Kyu Lee, Murukarthick Jayakodi, Nam-Hoon Kim, Hyun Jo Koo, Sang-Choon Lee, Yeon Jeong Kim, Sung Won Kwon, Tae-Jin Yang

**Affiliations:** ^1^Department of Plant Science, Plant Genomics and Breeding Institute, Research Institute of Agriculture and Life Sciences, College of Agriculture and Life Sciences, Seoul National UniversitySeoul, South Korea; ^2^College of Pharmacy and Research Institute of Pharmaceutical Sciences, Seoul National UniversitySeoul, South Korea; ^3^Crop Biotechnology Institute/GreenBio Science and Technology, Seoul National UniversityPyeongchang, South Korea

**Keywords:** *Panax ginseng*, cultivars, ginsenoside biosynthetic pathway, transcriptome, metabolome

## Abstract

*Panax ginseng* C.A. Meyer is a traditional medicinal herb that produces bioactive compounds such as ginsenosides. Here, we investigated the diversity of ginsenosides and related genes among five genetically fixed inbred ginseng cultivars (Chunpoong [CP], Cheongsun [CS], Gopoong [GO], Sunhyang [SH], and Sunun [SU]). To focus on the genetic diversity related to ginsenoside biosynthesis, we utilized *in vitro* cultured adventitious roots from the five cultivars grown under controlled environmental conditions. PCA loading plots based on secondary metabolite composition classified the five cultivars into three groups. We selected three cultivars (CS, SH, and SU) to represent the three groups and conducted further transcriptome and gas chromatography-mass spectrometry analyses to identify genes and intermediates corresponding to the variation in ginsenosides among cultivars. We quantified ginsenoside contents from the three cultivars. SH had more than 12 times the total ginsenoside content of CS, with especially large differences in the levels of panaxadiol-type ginsenosides. The expression levels of genes encoding squalene epoxidase (SQE) and dammarenediol synthase (DDS) were also significantly lower in CS than SH and SU, which is consistent with the low levels of ginsenoside produced in this cultivar. Methyl jasmonate (MeJA) treatment increased the levels of panaxadiol-type ginsenosides up to 4-, 13-, and 31-fold in SH, SU, and CS, respectively. MeJA treatment also greatly increased the quantity of major intermediates and the expression of the underlying genes in the ginsenoside biosynthesis pathway; these intermediates included squalene, 2,3-oxidosqualene, and dammarenediol II, especially in CS, which had the lowest ginsenoside content under normal culture conditions. We conclude that *SQE* and *DDS* are the most important genetic factors for ginsenoside biosynthesis with diversity among ginseng cultivars.

## Introduction

*Panax ginseng* is a medicinal plant that has long been used as a tonic agent throughout Asia, including Korea and China (Jia et al., 2009). Ginseng is rich in useful metabolites, such as polyacetylenes, polysaccharides, phenolic compounds, and terpenes. Triterpene saponins, also known as ginsenosides, are the major compounds in ginseng showing anti-oxidant, anti-inflammatory, and anti-cancer activity (Kim, 2012; Zhang et al., 2013). Dozens of ginseng accessions have been bred through pure line selection from three local landrace populations (Jakyung, Chungkyung, and Hwangsook) and registered as cultivated varieties (cultivars) in Korea (Kim et al., 2012). These cultivars exhibit different morphological and physiological characters (Kim et al., 2012; Lee et al., 2015), as well as different metabolite accumulation patterns (Ahn et al., 2008; Kim et al., 2009; Lee et al., 2011; Cho et al., 2012).

**FIGURE 1:**
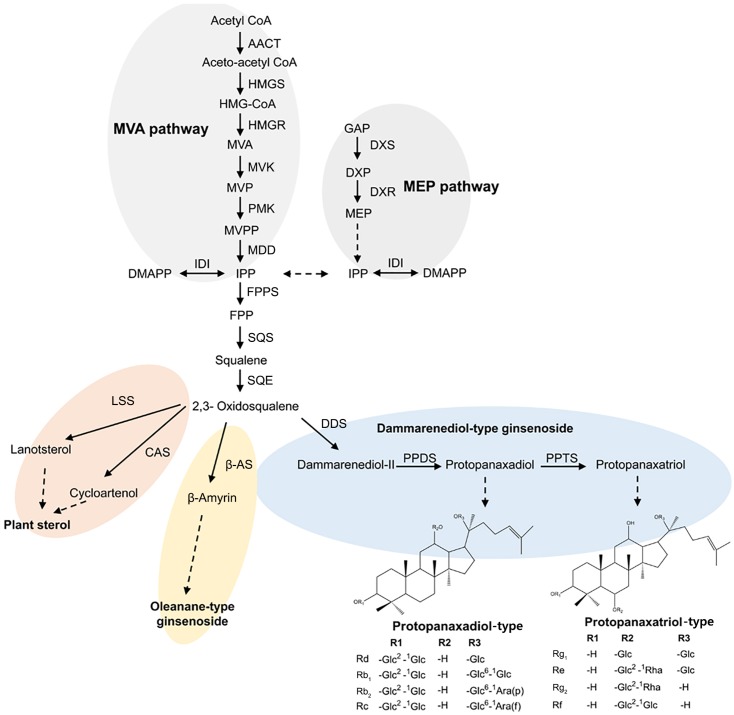
Schematic representation of the ginsenoside biosynthetic pathway in *Panax ginseng*; AACT, acetoacetyl CoA transferase; CAS, cycloartenol synthase; DMAPP, dimethylallyl diphosphate; DDS, dammarenediol synthase; DXP, 1-deoxy-D-xylulose 5-phosphate; DXR, 1-deoxy-D-xylulose 5-phosphate reductoisomerase; DXS, 1-deoxy-D-xylulose-5-phosphate synthase; FPP, farnesyl diphosphate; FPPS, farnesyl diphosphate synthase; GAP, glyceraldehyde 3-phosphate; HMGR, HMG-CoA reductase; HMGS, HMG-CoA synthase; IDI, isopentenyl-diphosphate delta-isomerase; IPP, isopentenyl diphosphate; LSS, lanosterol synthase; MEP, 2-C-methyl-D-erythritol 4-phosphate; MVK, mevalonate kinase; MVA, mevalonic acid; MDD, mevalonate diphosphate decarboxylase; MVP, mevalonic acid 5-phosphate; MVPP, 5-diphosphomevalonate; PMK, phosphomevalonate kinase; PPDS, protopanaxadiol synthase; PPTS, protopanaxatriol synthase; SQE, squalene epoxidase; SQS, squalene synthase; β-AS, β-amyrin synthase.

In *P. ginseng*, ginsenosides are mainly of the dammarane type, which are in turn classified into two types according to the number of hydroxyl groups: protopanaxadiol (PPD)-type ginsenosides with hydroxyl groups at positions C3, C12, and C20 and protopanaxatriol (PPT)-type ginsenosides with hydroxyl groups at positions C3, C6, C12, and C20 (Jung et al., 2014; Kim et al., 2014). A representative biosynthetic pathway for dammarane-type ginsenosides in ginseng is shown in **Figure 1**. Isopentenyl diphosphate (IPP) and dimethylallyl diphosphate (DMAPP) produced via the mevalonate (MVA) pathway undergo condensation and cyclization reactions catalyzed by farnesyl diphosphate synthase (FPPS), squalene synthase (SQS), and squalene epoxidase (SQE) (Kirby and Keasling, 2009; Thimmappa et al., 2014). Through this process, 2,3-oxidosqualene is generated and cyclized by dammarenediol II synthase (DDS) to produce dammarenediol II (Tansakul et al., 2006). Dammarenediol II is then hydroxylated by protopanaxadiol synthase (PgPPDS, CYP716A47) and converted to PPD (Han et al., 2011), which is further hydroxylated by protopanaxatriol synthase (PgPPTS, CYP716A53v2) to produce PPT (Han et al., 2012). Finally, PPD and PPT are glycosylated to produce various types of ginsenosides (Kim et al., 2014).

Candidate genes responsible for ginsenoside biosynthesis in the *Panax* genus have been identified in *P. ginseng* (Han et al., 2011, 2012; Jung et al., 2014; Kim et al., 2014; Jayakodi et al., 2015; Wang P. et al., 2015; Wei et al., 2015), *P. notoginseng* (Liu et al., 2015; Rai et al., 2016), and *P. vietnamensis* (Zhang et al., 2015). Transcriptome analysis of *P. notoginseng* identified several candidate unigenes encoding cytochrome P450 and glycosyltransferase and revealed that these genes were conserved across all *Panax* species (Rai et al., 2016). However, the regulatory mechanism for the ginsenoside biosynthesis pathway still remains unknown. Integrated omics analysis of the transcriptome and metabolome is an efficient tool for obtaining a precise understanding of biosynthetic pathways. This technique allows identification of novel functional genes and characterization of the regulatory mechanism and key factors of biosynthetic pathways (Saito et al., 2008; Mounet et al., 2009). The regulatory genes in the tryptophan (Dubouzet et al., 2007), flavonoid (Cho et al., 2016), flavanol (Yonekura-Sakakibara et al., 2008), and glucosinolate biosynthetic pathways (Hirai et al., 2004, 2007) have been explored using integrated analysis, and potential target genes have been identified.

In this study, we applied an integrated metabolomics and transcriptomics approach to explore the ginsenoside biosynthetic pathway using adventitious roots from five ginseng cultivars. Specifically, we compared the expression levels of genes involved in ginsenoside biosynthesis, the accumulation patterns of intermediates, and the composition and quantity of ginsenosides in adventitious roots with or without methyl jasmonate (MeJA) treatment. This analysis allowed us to uncover the dynamic regulatory mechanism for the ginsenoside biosynthetic pathway controlled by genetic factors and MeJA treatment.

## Materials and Methods

### Chemicals

Nine ginsenosides [Rg_1_, Re, Rf, Rg_2_(20*S*), Rg_2_(20*R*), Rb_1_, Rc, Rb_2_, and Rd], squalene, 2,3-oxidosqualene, mevalonolactone, and farnesol were purchased from Sigma–Aldrich (St. Louis, MO, United States). Dammarenediol II was kindly provided by Dr. Yong-Eui Choi (Kangwon National University, South Korea). Chemicals for tissue culture were purchased from Duchefa (Haarlem, The Netherlands), and the remaining chemicals were purchased from Sigma–Aldrich (St. Louis, MO, United States).

### Plant Materials

Adventitious root formation was induced from five *P. ginseng* cultivars (Chunpoong [CP], Cheongsun [CS], Gopoong [GO], Sunhyang [SH], and Sunun [SU]), and the roots were cultured as previously described (Jayakodi et al., 2014). Adventitious roots (42 days old) were treated with 200 μM MeJA. For metabolites profiling, the adventitious roots were harvested after 1 week of MeJA treatment, with five biological replicates for both non-treated and MeJA-treated adventitious roots. For quantification of gene expression using RT-PCR, adventitious roots were collected at 0, 12, 24, and 48 h after MeJA treatment, with three biological replicates. For transcriptome analysis, adventitious roots were harvested from plants without MeJA treatment, with three biological replicates. The adventitious roots were immediately stored at -80°C until use.

### Metabolome Analysis

#### Sample Preparation

For UPLC-ESI/MS analysis, 10 mg of lyophilized adventitious root tissue was sonicated with 1 mL of 70% methanol for 30 min at room temperature. The extracts were centrifuged at 13,000 ×*g* for 5 min, and the supernatants were filtered through a 0.2 μm PTFE syringe filter (Toyo Roshi Kaisha, Japan). For LC-UV/DAD analysis, 10 mg of lyophilized adventitious root tissue was sonicated three times using 2 mL of 70% methanol for 30 min at room temperature. The extracts were dried using N_2_ gas and resuspended in 200 μL of 80% methanol.

#### UPLC-ESI/MS Analysis

Metabolite profiling was carried out by ultra-performance liquid chromatography (ACQUITY UPLC system, Waters, United States) coupled with Q-TOF (micrOTOF-QII, Bruker Daltonics, Germany) using an ACQUITY BEH C18 column (2.1 mm × 100 mm, 1.7 μm, Waters, United States). The analysis was performed using a binary mobile phase consisting of A: water + 0.1% formic acid and B: acetonitrile + 0.1% formic acid. Separation was achieved under the following gradient conditions: pre-running with 100% A for 10 min, 100% A at 0 min, 65.5% A at 10 min, 52.5% A at 25 min, 20% A at 35 min, 0% A at 40 min and holding for 10 min. The flow rate was 0.2 mL/min, the column temperature was 40°C, and the injection volume was 5 μL.

The micrOTOF-QII was run in negative mode, and the source conditions were as follows: capillary -4.5 kV, nebulizer pressure at 1.2 bar, dry gas flow rate of 8 L/min, and dry gas temperature at 200°C. The ion transfer and collision stages were set as follows: funnel 1 RF 400 Vpp, funnel 2 RF 400 Vpp, hexapole RF 400 Vpp, quadrupole ion energy 15 eV, collision energy 10 eV, collision RF 400 Vpp, transfer time 100 μs, and pre-pulse storage 5 μs. High-purity nitrogen was used as the nebulizer and dry gas, and argon was used as the collision gas. Lithium formate was used as an internal standard, with a scan range from 50 to 1500 m/z.

#### LC-UV/DAD Analysis

The nine ginsenosides [Rg_1_, Re, Rf, Rg_2_(20*S*), Rg_2_(20*R*), Rb_1_, Rc, Rb_2_, and Rd] were quantified using an Agilent 1260 infinity HPLC system (Agilent, United States) with a discovery C18 column (2.1 mm × 100 mm, 5 μm, Sigma–Aldrich, United States). The mobile phase was composed of A: water + 0.1% formic acid and B: acetonitrile + 0.1% formic acid, as used for UPLC-ESI/MS. The flow rate was 0.3 mL/min. The gradient conditions were as follows: 80% A held for 20 min, followed by 70% A for 35 min, 55% A for 45 min, 10% A for 55 min, holding for 5 min, and a post-run with 80% A for 15 min. The UV detector was set at 203 nm, and the injection volume was 5 μL. For quantification, calibration curves of each ginsenoside were obtained, and intra- and interday precision and accuracy parameters were determined for validation (Supplementary Tables S1, S2).

#### GC-MS Analysis

The squalene, 2,3-oxidosqualene, farnesol, and dammarenediol II contents in CS, SH, and SU were analyzed by GC-MS as described previously (Kim et al., 2011; Lee et al., 2012). Briefly, KOH saponification-based extracts were analyzed using GCMS-QP2010 (Shimadzu, Japan) with a DB-5MS column (30 m × 0.25 mm, 0.25 μm, Agilent technologies, Germany). The carrier gas was helium, the flow rate was 1.0 mL/min, and the injection volume was 1 μL, in split mode (1:2). The mass detector was in electron impact mode of 70 eV, and the ion source temperature was 200°C. The mass detection range was 40–600 m/z. A single peak of each compound was identified using selected single ion monitoring mode and the retention times of authentic compounds. The areas of the compounds were normalized to that of the internal standard.

#### Data Processing and Multivariate Analysis

Raw data generated from UPLC-ESI/MS were processed using MZmine software version 2.10^1^. The data were subjected to peak deconvolution using a Savitzky-Golay filter and aligned through the RANdom Sample Consensus algorithm. The relative abundance of each peak was centered and scaled to unit variance, with base weight set at 1/standard deviation. The filtered peak lists were imported into SIMCA-P+ (version 12.0, Umetrics, Sweden), and principal component analysis (PCA) and partial least square-discriminant analysis (PLS-DA) were performed. In the PLS-DA model, metabolites with variable influence on the projection (VIP) value above 1 and *P*-value under 0.05 were selected for the VIP lists.

#### RNA Isolation and Transcriptome Sequencing Using the Illumina Platform

Total RNA was extracted from adventitious roots of cultivar SU using an RNeasy Plant Mini Kit (Qiagen, Germany) according to the manufacturer’s protocol. The quality and quantity of extracted RNA was checked using an ND-1000 (NanoDrop Technologies Inc., United States) and formaldehyde-agarose gel electrophoresis. An RNA-Seq library with a 300-bp insertion size was then constructed and sequenced on the Illumina sequencing platform (NextSeq 500, LabGenomics Co., South Korea). The three replicated RNA-Seq reads were deposited in the NCBI Sequence Read Archive^2^ (SRA) under accession numbers SRR2134236, SRR2134277, and SRR2134367. RNA-Seq data for adventitious roots from SH and CS were obtained from a previous study (Lee Y.S. et al., 2016).

#### Digital Expression Analysis of Genes Related to Ginsenoside Biosynthesis

For expression profiling among cultivars, candidate genes involved in ginsenoside biosynthesis were selected based on a previous report (Jayakodi et al., 2015). The expression levels of genes related to the ginsenoside biosynthetic pathway were determined based on fragments per kilobase of exon per million fragments (FPKM) values from RNA-seq data for CS, SH, and SU using RSEM (Li and Dewey, 2011). Genes showing significantly different expression among the three cultivars were identified by analysis of variance (ANOVA, *P*-value < 0.05) using R (version 3.1.0^3^) and the R packages mvtnorm (Genz et al., 2008) and multcomp (Hothorn et al., 2008). Mean FPKM values of the genes were converted to log2 values, which were used for visualization using the heatmap package in R. An integrative analysis between expression of the unigenes and the relative quantity of 21 metabolites was conducted with a bidirectional multivariate regression method based on orthogonal projection to latent structures (OPLS) (Bylesjö et al., 2007). The regression method of gene expression profiles (mean-centered, *x* variables) and metabolite profiles (mean-centered and Pareto-scaled, *y* variables) was analyzed using O2PLS model implemented in SIMCA tool (version 14.1, trial, Umetrics, Sweden).

#### RT-PCR Analysis

For RT-PCR analysis, cDNA was produced from total RNA of CS, SH, and SU using a SMART cDNA Synthesis kit (Clontech, Japan) according to the manufacturer’s protocol. The synthesized cDNA was diluted 1/10 and used as template for RT-PCR. The RT-PCR was performed on a LightCycler 480 (Roche, Germany) under the following thermal cycling conditions: 95°C for 5 min, followed by 40 cycles of 95°C for 15 s, 58°C for 10 s, and 72°C for 10 s. Primer sets for each gene were designed using Primer3 (Rozen and Skaletsky, 1999), and the specificity of the primer sets was confirmed by sequencing PCR amplicons on an ABI 3730 XL DNA Analyzer (Applied Biosystems, United States). All primer sets used in this study are listed in Supplementary Table S3.

## Results

### Secondary Metabolite Profiles in Adventitious Roots of Five Ginseng Cultivars

**FIGURE 2:**
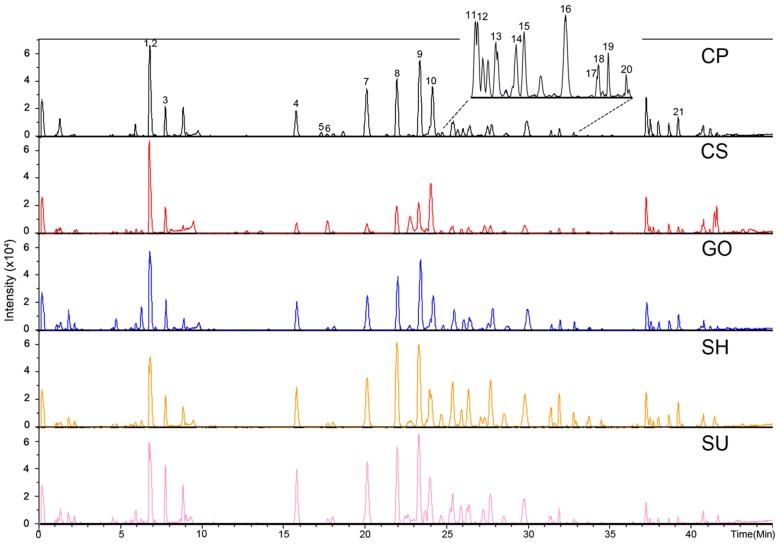
Representative chromatograph of adventitious roots from five ginseng cultivars (CP, CS, GO, SH, and SU) analyzed by UPLC-ESI/MS in negative mode; CP, Chunpoong; CS, Cheongsun; GO, Gopoong; SH, Sunhyang; and SU, Sunun. The identity of each peak is shown in **Table 1**.

We performed secondary metabolite profiling in adventitious root tissue from five ginseng cultivars (CP, CS, GO, SH, and SU) using UPLC-ESI/MS to investigate metabolic diversity among ginseng cultivars (**Figure 2**). A previously reported method using a reverse-phase column (Lee G.J. et al., 2016; Wang et al., 2016) was applied to selectively separate secondary metabolites including ginsenosides from adventitious root extracts. A total of 21 metabolites were identified via comparisons with authentic standards or data found in previous reports (Xie et al., 2008; Qi et al., 2012; MacCrehan and White, 2013). Among these, 16 metabolites with VIP values above 1 and *P*-value below 0.05 were characterized as biomarkers able to discriminate among adventitious roots from different ginseng cultivars. These biomarkers were PPD/PPT-type ginsenosides, except for four metabolites identified as chikusetsusaponin IVa, gypenoside XVII, calenduloside E, and an isomer of ginsenoside Ro (**Table 1**). These results indicate that PPD/PPT ginsenosides are the main contributors to the metabolic differences in adventitious roots among these cultivars.

**Table 1:** Metabolites in adventitious roots from five ginseng cultivars identified through UPLC-ESI/MS in negative mode (CP, Chunpoong; CS, Cheongsun; GO, Gopong; SH, Sunhyang; and SU, Sunun).

No.	RT (min)	m/z			Formula	Error (mDa)	Identified compound	VIP value	*P*-value	Reference
		Adduct ion	Exact	Measured						
1	6.8	[M-HCOO]^-^	845.49	845.48	C_42_H_72_O_14_	-6	Ginsenoside Rg_1_^a^	1.47	2.73E-30	STD^b^
2	6.9	[M-HCOO]^-^	991.55	991.54	C_48_H_82_O_18_	-5.7	Ginsenoside Re^a^	1.48	1.72E-30	STD^b^
3	7.7	[M-H]^-^	885.48	885.48	C_45_H_74_O_17_	-2.9	Malonyl ginsenoside Rg_1_	0.75	4.86E-11	MacCrehan and White, 2013
4	15.8	[M-H]^-^	799.48	799.48	C_42_H_72_O_14_	-0.7	Ginsenoside Rf^a^	1.29	2.78E-13	STD^b^
5	17.3	[M-H]^-^	1239.64	1239.64	C_59_H_100_O_27_	1.3	Notoginsenoside Fa	0.83	1.98E-25	Xie et al., 2008
6	17.7	[M-H]^-^	769.47	769.47	C_41_H_70_O_13_	0.9	Notoginsenoside R2^a^	1.35	1.63E-26	MacCrehan and White, 2013
7	20.1	[M-H]^-^	783.49	783.49	C_42_H_72_O_13_	1.8	Ginsenoside Rg_2_ (20S)/(20R) ^a^	1.45	6.58E-21	STD^b^
8	21.9	[M-H]^-^	1107.59	1107.59	C_54_H_92_O_23_	-1.1	Ginsenoside Rb_1_^a^	1.52	2.91E-18	STD^b^
9	23.3	[M-H]^-^	1193.59	1193.59	C_57_H_94_O_26_	-4.3	Malonyl ginsenoside Rb_1_^a^	1.55	7.30E-24	MacCrehan and White, 2013
10	23.9	[M-H]^-^	1077.58	1077.58	C_53_H_90_O_22_	-2.9	Ginsenoside Rc^a^	1.2	7.43E-28	STD^b^
11	25.2	[M-H]^-^	955.49	955.49	C_48_H_76_O_19_	-2.2	Ginsenoside Ro isomer	0.79	4.08E-17	Qi et al., 2012
12	25.4	[M-H]^-^	1163.58	1163.58	C_56_H_92_O_25_	-5.2	Malonyl ginsenoside Rb_2_/Rb_3_/Rc^a^	1.39	1.45E-25	Qi et al., 2012
13	26.3	[M-H]^-^	1077.58	1077.58	C_53_H_90_O_22_	-5.2	Ginsenoside Rb_2_^a^	1.15	8.24E-27	STD^b^
14	27.3	[M-H]^-^	925.48	925.47	C_47_H_74_O_18_	-0.6	Pseudoginsenoside RT_1_	0.83	2.77E-11	Qi et al., 2012
15	27.7	[M-H]^-^	1163.58	1163.58	C_56_H_92_O_25_	-4.2	Malonyl ginsenoside Rb_2_/Rb_3_/Rc^a^	1.28	1.29E-27	Qi et al., 2012
16	29.8	[M-H]^-^	793.44	793.44	C_42_H_66_O_14_	-1.4	Chikusetsusaponin IVa^a^	1.09	6.35E-17	Qi et al., 2012
17	31.3	[M-H]^-^	1163.58	1163.59	C_56_H_92_O_25_	-0.5	Malonyl ginsenoside Rb_2_/Rb_3_/Rc^a^	1.31	1.02E-21	Qi et al., 2012
18	31.4	[M-HCOO]^-^	991.55	991.55	C_48_H_82_O_18_	5.5	Ginsenoside Rd^a^	1.2	2.59E-14	STD^b^
19	31.9	[M-H]^-^	1031.54	1031.55	C_51_H_84_O_21_	3.1	Malonyl ginsenoside Rd isomer^a^	1.12	1.27E-19	Qi et al., 2012
20	32.8	[M-HCOO]^-^	991.55	991.56	C_48_H_82_O_18_	3.1	Gypenoside XVII	0.97	1.19E-28	Qi et al., 2012
21	39.2	[M-H]^-^	631.38	631.38	C_36_H_56_O_9_	-4.2	Calenduloside E^a^	1.21	3.48E-13	Qi et al., 2012

*^a^Characterized biomarker metabolites (VIP value > 1.0 and P-value < 0.05).*

*^b^Identified metabolites using authentic compounds.*

**FIGURE 3:**
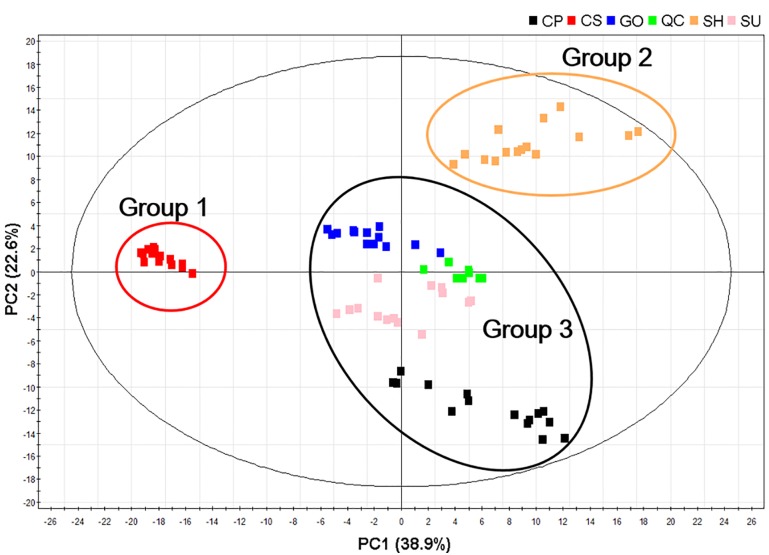
Principal component analysis (PCA) score plots of adventitious roots from five ginseng cultivars (CP, CS, GO, SH, and SU) analyzed by UPLC-ESI/MS in negative mode; CP, Chunpoong; CS, Cheongsun; GO, Gopoong; SH, Sunhyang; and SU, Sunun.

We subjected the UPLC-ESI/MS data to multivariate analysis to visualize the metabolic differences among cultivars. The five ginseng cultivars were divided into three groups according to the PCA loading plots (R^2^X: 0.809 and d Q^2^: 0.825): Group 1: CS; Group 2: SH; and Group 3: SU, CP, and GO. This grouping indicated that the accumulation pattern of metabolites varied among cultivars, but that some cultivars shared similar metabolic profiles (**Figure 3**).

**FIGURE 4:**
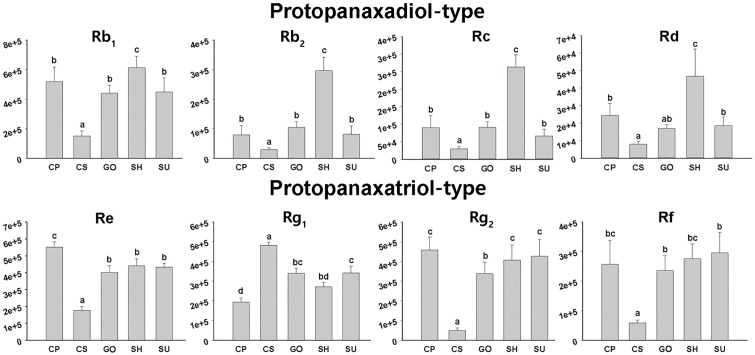
Relative abundances of eight ginsenosides in adventitious roots from five ginseng cultivars (CP, CS, GO, SH, and SU) analyzed by UPLC-ESI/MS in negative mode; CP, Chunpoong; CS, Cheongsun; GO, Gopoong; SH, Sunhyang; and SU, Sunun.

UPLC-ESI/MS analysis also revealed that the relative abundance of representative ginsenosides in adventitious roots differed among the five ginseng cultivars (**Figure 4**). SH roots were rich in PPD-type ginsenosides such as Rb_1_, Rb_2_, Rc, and Rd compared to the other cultivars, although the amount of PPT-type ginsenoside in SH was comparable to that in other cultivars except CS. CS showed the lowest amounts of both PPD and PPT ginsenosides, although it was relatively rich in Rg_1_. The abundances of PPD-type and PPT-type ginsenosides were not significantly different among cultivars CP, GO, and SU.

### Transcriptome Analysis of Adventitious Roots from CS, SH, and SU

UPLC-ESI/MS analysis revealed differences in the accumulation patterns of metabolites among ginseng cultivars, suggesting that the ginsenoside biosynthetic pathway might be differentially regulated in these cultivars (**Table 1** and **Figure 3**). We selected one cultivar to represent each group in the PCA loading plots (CS, SH, and SU; **Figure 3**) and compared these three cultivars via transcriptome analysis (**Table 2**). We obtained transcriptome data for adventitious roots from CS and SH from a previous study (Lee Y.S. et al., 2016) and generated transcriptome data for SU in the current study. An average of 15 million (M) reads from three replicates were produced from SU adventitious roots. After trimming, an average of 14 M reads was subsequently used for expression profiling of genes involved in the ginsenoside biosynthetic pathway.

**Table 2:** Summary of transcriptome data generated from adventitious roots of *Panax ginseng* cultivars CS, SH, and SU (CS, Cheongsun; SH, Sunhyang; and SU, Sunun).

Adventitious root sample (SRA accession number)	Raw data		QC filtered data	
	Total no. of reads	Length (bp)	Total no. of reads	Length (bp)
CS, replicate 1^a^ (SRR2132332)	16,731,664	2,321,621,040	15,291,398	2,115,447,628
CS, replicate 2^a^ (SRR1688723)	14,306,820	1,977,938,763	12,831,592	1,766,225,247
CS, replicate 3^a^ (SRR2132333)	14,801,822	2,056,937,756	13,407,302	1,857,154,376
SH, replicate 1^a^ (SRR1688724)	17,526,160	2,446,652,196	15,710,392	2,185,318,113
SH, replicate 2^a^ (SRR2132380)	16,947,000	2,354,285,233	15,409,700	2,134,138,490
SH, replicate 3^a^ (SRR2132382)	16,426,236	2,281,803,413	14,810,282	2,050,279,385
SU, replicate 1 (SRR2134236)	15,542,974	2,155,072,375	14,066,330	1,943,820,582
SU, replicate 2 (SRR2134277)	15,770,684	2,187,522,302	14,314,738	1,979,136,578
SU, replicate 3 (SRR2134367)	15,461,854	2,160,894,730	13,846,510	1,928,707,399
Total	186,981,726	22,553,682,825	172,796,402	20,550,270,711

*^a^Transcriptomes of CS and SH were obtained from a previous report (Lee Y.S. et al., 2016).*

### Comparison of Ginsenoside Biosynthesis Pathways in Adventitious Roots from CS, SH, and SU

We previously identified 23 candidate unigenes related to the MVA and triterpene biosynthetic pathways from the *P. ginseng* root transcriptome database (Jayakodi et al., 2015). To identify genes associated with the differences in ginsenoside contents among cultivars, we analyzed the expression levels of the 23 unigenes based on FPKM values and compared the expression patterns among cultivars (**Table 3**).

**Table 3:** FPKM values of unigenes involved in the ginsenoside biosynthetic pathway in adventitious roots of CS, SH, and SU (CS, Cheongsun; SH, Sunhyang; and SU, Sunun).

Pathway	Gene name	Candidate unigene	FPKM		
			CS	SH	SU
Mevalonate pathway	*AACT*	Pg_Root114975_c0_seq10	15.87 ± 10.88	25.5 ± 9.11	19.24 ± 7.59
	*HMGS*	Pg_Root104689_c0_seq1	48.13 ± 13.29	62.2 ± 7.43	53.29 ± 9.23
	*HMGR*	Pg_Root112393_c0_seq27	4.57 ± 6.46	0	0
	*HMGR*	Pg_Root123699_c0_seq7	0.11 ± 0.16	0.04 ± 0.06	0.17 ± 0.13
	*HMGR*	Pg_Root123699_c0_seq4	4.72 ± 1.44	10.55 ± 7.81	13.63 ± 0.55
	*MVK*	Pg_Root113545_c0_seq1	15.18 ± 5.56	6.47 ± 0.91	14.15 ± 3.7
	*PMK*	Pg_Root127818_c1_seq23	27.01 ± 4.2	15.8 ± 5.18	21.67 ± 7.77
	*PMK*	Pg_Root127818_c1_seq9	3.57 ± 5.05	9.79 ± 7.35	9.2 ± 13.02
	*PMK*	Pg_Root101855_c0_seq2	0	3.15 ± 2.24	6.91 ± 6.34
	*PMK*	Pg_Root101855_c0_seq9	2.57 ± 3.64	0	0.87 ± 1.23
	*MDD*	Pg_Root126585_c2_seq1	21.89 ± 6.6	27.93 ± 5.51	32.42 ± 12.77
	*IDI*	Pg_Root110920_c0_seq2	74.68 ± 9.86	81.49 ± 8.81	52.35 ± 18.11
Triterpene biosynthesis pathway	*FPPS*	Pg_Root127352_c0_seq7	12.96 ± 2.33	4.92 ± 4.7	12.15 ± 4.85
	*FPPS*	Pg_Root127352_c0_seq34	0	8.66 ± 12.24	0
	*FPPS^∗^*	Pg_Root127352_c0_seq41	18.02 ± 3.33ˆa	10.33 ± 7.4ˆa	27.13 ± 3.33ˆb
	*SQS*	Pg_Root122824_c0_seq14	30.01 ± 5.78	37.73 ± 4.06	26.69 ± 9.2
	*SQE*	Pg_Root125745_c1_seq5	5.45 ± 2.91	4.13 ± 1.47	5.97 ± 1.94
	*SQE^∗^*	Pg_Root104873_c0_seq1	11.24 ± 7.8ˆa	39.42 ± 12.87ˆc	15.07 ± 5.53ˆab
	*DDS^∗^*	Pg_Root126438_c1_seq1	12.5 ± 2.79ˆa	82.34 ± 27.84ˆb	99 ± 4.07ˆb
	*β-AS*	Pg_Root120424_c0_seq1	0.63 ± 0.89	2.41 ± 1.15	2.83 ± 2.57
	*β-AS^∗^*	Pg_Root120424_c0_seq15	0.66 ± 0.93ˆa	2.71 ± 1.34ˆa	4.04 ± 1.74ˆb
	*PPDS^∗^*	Pg_Root123943_c0_seq1	40.56 ± 15.36ˆa	59.18 ± 15.78ˆa	84.42 ± 8.82ˆb
	*PPTS*	Pg_Root91292_c0_seq1	101.35 ± 48.73	72.3 ± 10.28	117.19 ± 10.06

*^∗^Significantly differentially expressed unigenes among adventitious roots from CS, SH, and SU are marked with an asterisk (P < 0.05), and significant differences are represented by different letters. CS, Cheongsun; SH, Sunhyang; SU, Sunun. The full names of genes are shown in **Figure 1**.*

Among the seven candidate genes in the MVA pathway, unigene Pg_Root110920_c0_seq2 (encoding IDI) showed the highest expression while unigene Pg_Root123699_c0_seq7 (encoding HMGR) showed the lowest expression in adventitious roots from CS, SH, and SU. The expression levels of most unigenes in MVA biosynthesis were not significantly different among cultivars (**Table 3**). By contrast, the expression levels of unigenes involved in the triterpene biosynthesis pathway differed significantly (**Table 3**). Unigene Pg_Root127352_c0_seq41 (encoding FPPS) was highly expressed in SU. Unigene Pg_Root104873_c0_seq1 (encoding SQE) and unigene Pg_Root126438_c1_seq1 (encoding DDS) were expressed at extremely low levels in CS compared to SH and SU. The expression level of each gene was correlated with the relative amounts of intermediates and total ginsenosides in adventitious roots of the three cultivars (**Figure 4**). Unigene Pg_Root123943_c0_seq1 (encoding PPDS) was the most highly expressed in SU whereas unigene Pg_Root91292_c0_seq1 (encoding PPTS) was highly expressed in all cultivars (**Table 3**). Unigene Pg_Root120424_c0_seq15 (encoding β-amyrin synthase; β-AS) showed different expression among cultivars but its expression level was low compared to that of the *DDS* unigene (Pg_Root126438_c1_seq1).

The integration of gene expression with metabolite data by O2PLS model resulted in co-variation of 62% of transcripts with 80% of the metabolites. A pairwise Pearson correlations for those transcripts and metabolites showed a strong correlation (*r* > 0.7) with more than seven metabolites between unigene Pg_Root104873_c0_seq1 (encoding SQE) and Pg_Root126438_c1_seq1 (encoding DDS) (Supplementary Figure S1).

### Ginsenoside Contents Are Altered after MeJA Treatment in Adventitious Roots from CS, SH, and SU

Methyl jasmonate treatment upregulates ginsenoside biosynthesis in *P. ginseng* (Yendo et al., 2010). We investigated the effects of MeJA treatment on ginsenoside biosynthesis in different ginseng cultivars to understand the underlying genetic factors (**Table 4**). LC-UV-DAD analysis revealed that the three representative ginseng cultivars had different responses to MeJA treatment. In all three cultivars, the contents of PPD-type ginsenosides under MeJA treatment were higher than those of PPT-type ginsenosides. After 7 days of MeJA treatment, PPD-type ginsenoside levels increased 4.14- (SH), 13.15- (SU), and 31.44- (CS) fold, whereas PPT-type ginsenoside levels increased 1.34- (SH), 4.07- (SU), and 4.00- (CS) fold (**Table 4**).

**Table 4:** Ginsenoside contents in adventitious roots of CS, SH, and SU following 7 days of methyl jasmonate (MeJA) treatment, as analyzed by LC-UV-DAD (CS, Cheongsun; SH, Sunhyang; and SU, Sunun).

Ginsenosides (μg/mg)	CS			SH			SU		
	Control	+MJ	Fold	Control	+MJ	Fold	Control	+MJ	Fold
Rb_1_	0.15 ± 0.06^c^	2.58 ± 0.28^b^	17.38	1.21 ± 0.06^a^	4.99 ± 0.73^a^	4.13	0.43 ± 0.07^b^	5.42 ± 0.47^a^	12.61
Rb_2_	0.03 ± 0.10^c^	1.01 ± 0.10^c^	32.92	0.90 ± 0.31^a^	2.34 ± 0.31^a^	2.60	0.11 ± 0.16^b^	1.71 ± 0.16^b^	15.71
Rc	0.14 ± 0.02^c^	4.22 ± 0.52^c^	30.43	2.23 ± 0.04^a^	8.62 ± 0.97^a^	3.86	0.61 ± 0.05^b^	7.17 ± 0.50^b^	11.85
Rd	0.04 ± 0.01^c^	3.34 ± 0.51^a^b	90.32	0.36 ± 0.04^a^	3.48 ± 0.63^a^	9.78	0.13 ± 0.02^b^	2.41 ± 0.32^b^	18.97
Total panaxadiol	0.35 ± 0.09^c^	11.15 ± 1.33^c^	31.44	4.7 ± 0.16^a^	19.42 ± 2.57^a^	4.14	1.27 ± 0.12^b^	16.71 ± 1.16^b^	13.15
Re	0.24 ± 0.01^c^	1.72 ± 0.32^c^	7.05	3.4 ± 0.20^a^	5.60 ± 1.22^a^	1.65	1.16 ± 0.03^b^	7.37 ± 0.80^b^	6.36
Rg_1_	0.96 ± 0.07^a^	3.08 ± 0.16^a^	3.19	2.43 ± 0.09^b^	2.26 ± 1.05^a^	0.93	1.24 ± 0.05^b^	2.27 ± 0.49^a^	1.83
Rg_2_ (20*S*)-form	0.02 ± 0.00^c^	0.05 ± 0.00^b^	2.50	0.10 ± 0.01^a^	0.07 ± 0.02^a^b	0.69	0.03 ± 0.00^b^	0.08 ± 0.01^a^	2.29
Rg_2_ (20*R*)-form	0.02 ± 0.00^c^	0.17 ± 0.02^b^	8.24	0.52 ± 0.07^a^	0.69 ± 0.17^a^	1.33	0.14 ± 0.01^b^	0.88 ± 0.18^a^	6.39
Rf	0.06 ± 0.01^c^	0.20 ± 0.01^b^	3.30	0.23 ± 0.02^a^	0.32 ± 0.05^a^	1.40	0.13 ± 0.01^b^	0.39 ± 0.05^a^	3.00
Total panaxatriol	1.31 ± 0.07^c^	5.22 ± 0.35^b^	4.00	6.68 ± 0.28^a^	8.94 ± 2.20^a^	1.34	2.70 ± 0.08^b^	10.99 ± 1.41^a^	4.07
Total ginsenosides	1.66 ± 0.19^a^	16.37 ± 9.86^a^	9.86	11.38 ± 0.59^c^	28.36 ± 2.49^b^	2.49	3.97 ± 0.25^b^	27.70 ± 6.98^b^	6.98

*^∗^Significantly differentially expressed unigenes among adventitious roots of CS, SH, and SU are represented by different letters (CS, Cheongsun; SH, Sunhyang; SU, Sunun).*

Ginsenoside levels responded most strongly to MeJA treatment in CS, which had the lowest ginsenoside levels of the three cultivars under normal conditions, as well as the lowest expression of ginsenoside biosynthesis genes. In MeJA-treated CS adventitious roots, levels of Rd, a PPD-type ginsenoside, increased 90.32-fold, while the levels of other PPD-type ginsenosides, such as Rb_1_, Rc, and Rb_2_, increased 17.38-, 30.43-, and 32.92-fold, respectively. SH, the most ginsenoside-rich cultivar under normal conditions, was less responsive to MeJA treatment compared to CS and SU (**Table 4**).

### Gene Expression and Quantity of Intermediates in Ginsenoside Biosynthesis under MeJA Treatment

**FIGURE 5:**
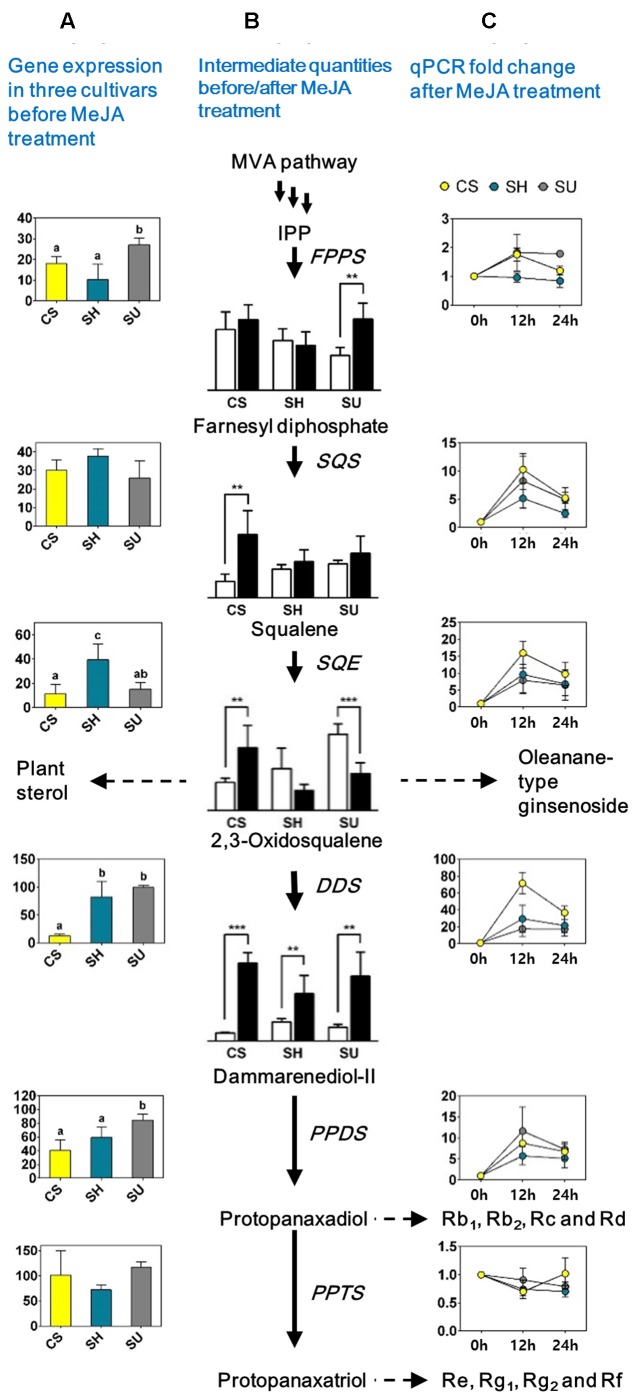
Quantification of gene expression levels and levels of intermediates involved in the ginsenoside biosynthetic pathway in adventitious roots with or without methyl jasmonate (MeJA) treatment in three ginseng cultivars; CS, Cheongsun; SH, Sunhyang; and SU, Sunun. **(A)** Expression levels of the genes among the three ginseng cultivars. The FPKM values (*Y*-axis) of each gene in **Table 3** were compared among cultivars. **(B)** Ginsenoside biosynthesis pathway and quantity of intermediates among three cultivars before (white bars) and after MeJA treatment (black bars). Relative abundances of intermediates are represented in the bar graphs; significant differences are indicated by asterisks: ^∗^ *P* < 0.05; ^∗∗^ *P* < 0.01 (*t*-test). **(C)** Expression (fold change) of each gene before and after MeJA treatment in adventitious roots of the three ginseng cultivars. Fold changes (*Y*-axis) in gene expression levels at 12 and 24 h (*X*-axis) compared to 0 h (analyzed by RT-PCR) are shown in the line graphs.

To identify the genes and intermediates associated with changes in ginsenoside contents in the three cultivars following MeJA treatment, we analyzed the expression levels of genes and the contents of intermediates related to the triterpene biosynthetic pathway in MeJA-treated CS, SH, and SU adventitious roots (**Figure 5**).

Gene expression analysis via RT-PCR revealed differences in the expression patterns of genes involved in the MVA pathway and the triterpene biosynthetic pathway (**Figure 5** and Supplementary Table S4). Among genes in the MVA pathway, only *AACT* expression significantly increased after MeJA treatment (Supplementary Table S4). By contrast, most genes corresponding to the triterpene biosynthetic pathway were significantly induced by MeJA treatment, with different levels of increase detected among cultivars, indicating that the triterpene biosynthetic pathway could be responsible for the variations in ginsenoside accumulation among different genotypes of *P. ginseng*. In particular, the expression of *DDS* dramatically increased (17.62- to 71.83-fold) at 12 h post-elicitation in all cultivars, and *SQE* (7.9- to 15.97-fold) and *SS* (5.16- to 10.32-fold) were also significantly induced. Notably, the changes in expression of all three genes were highest in the cultivar with the lowest ginsenoside content, CS (**Figure 5**).

GC-MS analysis revealed that the relative abundance of intermediate compounds, from squalene to dammarenediol II, following MeJA treatment increased the most in CS **(Figure 5**). Comparative analysis of adventitious roots among the three cultivars with or without MeJA treatment suggested that squalene to dammarenediol II might be the major limiting step that affects the variation in ginsenoside content among ginseng cultivars. Among these, *DDS* likely plays the most critical role in ginsenoside biosynthesis and accumulation.

## Discussion

### Metabolic Profiles in Ginseng Adventitious Roots

Ginsenoside profiles and accumulation patterns can differ based on genotype (Ahn et al., 2008; Kim et al., 2009) and growth conditions (Matkowski, 2008). In this study, we focused on the genetic component of ginsenoside accumulation by using five genetically fixed inbred cultivars, CP, CS, GO, SH, and SU (Kwon et al., 2001; Ahn et al., 2008; Lee et al., 2008) and isolating ginsenosides from *in vitro* cultured adventitious roots grown under a controlled environment. We quantified the variation among cultivars in the contents of ginsenosides and intermediates in the ginsenoside biosynthesis pathway as well as the relevant differences in expression of the underlying genes.

We characterized 21 ginsenosides, 15 of which were PPD/PPT-type ginsenosides. Among these PPD/PPT-type ginsenosides, six metabolites were identified as malonyl-ginsenosides (**Table 1**). Malonylation, a modification process that occurs widely in plants, takes place on the sugar moieties of phytohormones, xenobiotic compounds, and several secondary metabolites (Muth et al., 2008; Kochkin et al., 2013). Malonylation plays a role in improving the solubility of target compounds and facilitating their transport into storage organelles such as vacuoles (Taguchi et al., 2010). In the current study, malonyl-conjugated PPD-type ginsenosides accounted for five of the six identified malonyl-ginsenosides, which is consistent with a previous report that malonylation occurs more often in PPD-type ginsenosides than in PPT-type ginsenosides of *Panax* (Kochkin et al., 2013). Thus, some PPD-type ginsenosides that accumulate in adventitious roots are malonylated and, therefore, might be stored in organelles such as vacuoles.

The ginsenosides present in adventitious roots had different accumulation patterns in different cultivars. PCA loading plots revealed that that five ginseng cultivars could be divided into three groups according to their secondary metabolite profiles, although the PCA plot based on ginsenoside profile did not correlate precisely with that based on primary metabolite profiles (Lee Y.S. et al., 2016). Notably, however, the CS cultivar exhibited distinct metabolic accumulation in both the PCA loading plots based on primary metabolite profiles (Lee Y.S. et al., 2016) and those based on secondary metabolite profiles (**Figure 3**). These results indicate that the ginsenoside pathway is likely distinct in CS adventitious roots compared to other ginseng cultivars.

### Dammarane-Type Ginsenoside Synthesis Rapidly Increases in Response to MeJA Treatment

Jasmonic acid and MeJA are signaling compounds related to plant defense (Derksen et al., 2013). They also have been used as important plant-derived elicitors for elevated production of secondary metabolites in various plant species (Ali et al., 2006; Han et al., 2006; Lee et al., 2013; Wang J. et al., 2015). Oxidosqualene, a precursor for dammarenediol synthase, is present at a branch point in the triterpene biosynthesis pathway. This compound is cyclized by various oxidosqualene cyclases, such as lanosterol synthase (LSS), cycloartenol synthase (CAS), β-AS, and DDS to produce phytosterols and ginsenosides (Thimmappa et al., 2014). LSS and CAS catalyze the conversion of oxidosqualene to lanosterol and cycloartenol, respectively, which is the first step for entry into phytosterol biosynthesis (Suzuki et al., 2006; Ohyama et al., 2009). The expression levels of *LSS* and *CAS* were constant or decreased in response to MeJA treatment in *Centella asiatica* (Bonfill et al., 2011) and also in *P. ginseng* (Han et al., 2006), suggesting that the phytosterols in ginseng play a fundamental role in plant growth and development rather than in defense mechanisms involving MeJA. In addition, the expression of *β-AS*, encoding an enzyme that produces β-amyrin from oxidosqualene during the first committed step of oleanane-type ginsenoside biosynthesis (Suzuki et al., 2006; Tansakul et al., 2006), is not significantly altered under MeJA treatment (Han et al., 2006). By contrast, we found that both the contents of dammarane-type ginsenosides (especially PPD-type) and the expression of *DDS* significantly increased after MeJA treatment (**Figure 5** and **Table 4**). This result suggests that the biological activities of dammarane-type ginsenosides are more closely involved in defense in ginseng than are those of oleanane-type ginsenosides. The differential regulation of dammarane-type ginsenoside biosynthesis in the ginseng cultivars we examined might influence the properties of these cultivars in terms of plant defense responses.

### Dynamic Alteration of Ginsenoside Biosynthesis via MeJA Treatment

In the absence of MeJA treatment, the ginsenoside contents were considerably lower in adventitious roots of CS as compared to the other cultivars (**Table 4** and **Figure 4**), which correlated well with the lower expression in CS of genes that function in the pathway from squalene to dammarenediol II (**Table 3**). In addition, the ginsenoside contents significantly increased in CS adventitious roots upon MeJA treatment; PPD-type ginsenoside levels were increased 17- to 90-fold, and the expression levels of genes such as *SQS, SQE*, and *DDS*, as well as the contents of intermediates such as squalene, 2,3-oxidosqualene, and dammarenediol II, were highly elevated (**Figure 5**). Remarkably, the expression level of *DDS* was upregulated 71-fold in CS in response to MeJA treatment and the relative dammarenediol II level in CS adventitious roots became comparable to those in SH and SU (**Figure 5**). In the case of CS, our data indicate that the low ginsenoside contents in adventitious roots are likely related to the low expression levels of *SQE* and *DDS*. The factors that underlie the low expression of these genes in CS are yet not known, but might be directly related to differences in function of the genes, such as unknown transcription factors of *DDS*.

The dramatic increase in *DDS* expression under MeJA treatment coincided with the rapid increase in ginsenoside contents, especially in CS adventitious roots. Indeed, the content of dammarane-type ginsenosides is decreased by 84.5% in the roots of transgenic ginseng plants with downregulated *DDS* expression (Han et al., 2006). These findings suggest that the route from squalene to dammarenediol II is a major factor responsible for the diversity of the flux through the ginsenoside biosynthetic pathway in various ginseng cultivars and that DDS might play a key role in the upregulation of ginsenoside biosynthesis.

## Conclusion

We investigated the diversity of the ginsenoside biosynthetic pathway among adventitious roots from five ginseng cultivars using integrated transcriptomic and metabolomic analysis. We elucidated the variation in the ginsenoside biosynthetic pathway by comparing the expression levels of genes, the relative abundance of intermediate compounds, and the ginsenoside contents in adventitious roots of five genetically fixed inbred ginseng cultivars grown in a controlled environment. We also examined the intrinsic variation in ginsenoside contents and gene expression levels in the cultivars before and after MeJA treatment. Major genes in the triterpene biosynthesis pathway, including *SQE* and *DDS*, were found to play an important role in regulating ginsenoside biosynthesis, as well as in the variation of ginsenoside contents. The genes responsible for the metabolic diversity of these ginseng cultivars are good candidates to be utilized for the genetic improvement of this important medicinal plant.

## Author Contributions

T-JY designed the research and organized the manuscript. YSL and H-SP conducted adventitious root culture and transcriptome analysis. D-KL and SWK conducted metabolome analysis. YSL, H-SP, D-KL, MJ, N-HK, HJK, S-CL, and YJK conducted analysis of integrated bioinformatics data. YSL, H-SP, and T-JY wrote and revised the manuscript. All authors approved the final manuscript.

## Conflict of Interest Statement

The authors declare that the research was conducted in the absence of any commercial or financial relationships that could be construed as a potential conflict of interest.

## Footnotes

1 http://mzmine.sourceforge.net/

2 http://www.ncbi.nlm.nih.gov/Traces/sra

3 www.R-project.org
