# Supplementary material for: Integrated Transcriptomic and Metabolomic Analysis of Five Panax ginseng Cultivars Reveals the Dynamics of Ginsenoside Biosynthesis
Source: Front Plant Sci. 2017 Jun 19;8:1048. doi: 10.3389/fpls.2017.01048 (PMC5474932; doi:10.3389/fpls.2017.01048)
Supplement: Supplementary file 1 [file Table_1.DOCX]

Table S1. Validation of nine ginsenosides through linearity regression analysis

|  | Rg_1_ | Re | Rb_1_ | Rc | Rd | Rb_2_ | Rf | Rg_2_(20*S*) | Rg_2_(20*R*) |
| --- | --- | --- | --- | --- | --- | --- | --- | --- | --- |
| R^2^ | 0.992 | 0.992 | 0.987 | 0.993 | 0.994 | 0.995 | 0.99 | 0.996 | 0.994 |
| Slope (ppm/area) | 5.0 | 3.4 | 3.2 | 3.2 | 4.2 | 3.6 | 4.8 | 6.8 | 4.5 |
| Calibration range (ppm) | 50-300 | 50-300 | 50-300 | 50-300 | 50-300 | 10-50 | 10-50 | 10-50 | 10-50 |
| LOD (ppm) | 0.9 | 0.1 | 0.3 | 0.7 | 0.1 | 0 | 1.4 | 0.5 | 0.6 |
| LOQ (ppm) | 2.7 | 0.4 | 0.8 | 2.2 | 0.3 | 0.1 | 4.3 | 1.6 | 1.8 |

LOD: Limit of detection; LOQ: Limit of quantification
