# Supplementary material for: Integrated Transcriptomic and Metabolomic Analysis of Five Panax ginseng Cultivars Reveals the Dynamics of Ginsenoside Biosynthesis
Source: Front Plant Sci. 2017 Jun 19;8:1048. doi: 10.3389/fpls.2017.01048 (PMC5474932; doi:10.3389/fpls.2017.01048)
Supplement: Supplementary file 2 [file Table_2.DOCX]

Table S2. Intra- and interday precision and accuracy parameters of nine ginsenosides

|  |  | Concentration | Rg1 | Re | Rb1 | Rc | Rd | Rb2 | Rf | Rg2S | Rg2R |
| --- | --- | --- | --- | --- | --- | --- | --- | --- | --- | --- | --- |
| Intraday (%) | Precision | Low | 0.8 | 0.5 | 0.8 | 1.3 | 1.3 | 5.5 | 3.2 | 5.0 | 7.5 |
|  |  | Middle | 1.4 | 0.2 | 0.6 | 0.5 | 1.0 | 4.6 | 2.9 | 2.3 | 5.4 |
|  |  | High | 3.2 | 2.6 | 1.3 | 3.3 | 1.2 | 4.3 | 4.9 | 1.8 | 3.7 |
|  | Accuracy | Low | 108-110 | 102-103 | 101-103 | 99-102 | 100-103 | 94-107 | 108-115 | 98-109 | 93-110 |
|  |  | Middle | 92-95 | 98-98 | 101-102 | 103-104 | 100-102 | 90-99 | 89-95 | 89-94 | 83-93 |
|  |  | High | 92-99 | 96-102 | 98-101 | 98-106 | 98-100 | 104-115 | 109-123 | 107-112 | 100-108 |
| Interday (%) | Precision | Low | 1.0 | 1.9 | 0.7 | 1.2 | 1.7 | 1.7 | 13.4 | 6.2 | 7.8 |
|  |  | Middle | 1.3 | 0.5 | 1.4 | 1.0 | 0.1 | 6.8 | 5.7 | 4.3 | 9.0 |
|  |  | High | 1.2 | 2.0 | 0.4 | 1.0 | 0.7 | 6.4 | 4.8 | 2.0 | 1.5 |
|  | Accuracy | Low | 107-109 | 99-102 | 100-102 | 99-101 | 98-101 | 101-105 | 95-122 | 104-119 | 83-99 |
|  |  | Middle | 104-107 | 101-103 | 101-105 | 100-102 | 99-100 | 96-112 | 95-108 | 99-110 | 90-111 |
|  |  | High | 99-102 | 97-102 | 100-101 | 100-103 | 98-100 | 102-119 | 101-113 | 109-114 | 115-119 |
