# Supplementary material for: Integrated Transcriptomic and Metabolomic Analysis of Five Panax ginseng Cultivars Reveals the Dynamics of Ginsenoside Biosynthesis
Source: Front Plant Sci. 2017 Jun 19;8:1048. doi: 10.3389/fpls.2017.01048 (PMC5474932; doi:10.3389/fpls.2017.01048)
Supplement: Supplementary file 3 [file Table_3.DOCX]

Table S3. Primer list for RT-PCR

| Target gene | Direction | Sequence (5'-3') |
| --- | --- | --- |
| *AACT* | F | TGCAAATTTGGGTCAGGCTC |
| *AACT* | R | GAGACCCCTTCCTTGCTTCT |
| *HMGS* | F | AAGCTATGCTAGCCGTGATCTTG |
| *HMGS* | R | CCTTCACGAAGATGGAATGAAA |
| *HMGR* | F | TGCTGCCAATATCGTCTCTG |
| *HMGR* | R | GAGCTCTCCAGCCAAAACTG |
| *MVK* | F | AAGCCCACTTGTTCACCAAC |
| *MVK* | R | CCAGGGATATAAGCCTGCAA |
| *PMK* | F | GTTGGAGGCCAGCCATTAGA |
| *PMK* | R | TTTCTTGACAGCACCGACA |
| *MDD* | F | AGGAAGTTGTACCGAAACGCAT |
| *MDD* | R | CAGAACGGTTCCATTTCTCTACG |
| *FPPS* | F | CAGAGGTCAACCCTGTTGGT |
| *FPPS* | R | CAGGCCACTGGAAGGTAAAA |
| *SQS* | F | TTGCGAGGTCCTGCTATCTT |
| *SQS* | R | CTTTTGGACAGGGTTCCAGA |
| *SQE* | F | CTTTCAATATGCGCCATCCT |
| *SQE* | R | TGGCCCTTGAGAACAAATTC |
| *DDS* | F | ACCGCCGTTGAGATTAGATG |
| *DDS* | R | TATAGGATCCCCATCCACCA |
| *PPDS* | F | ATGCATCTTCCATCCCTTTG |
| *PPDS* | R | GTACGGACCGGACGACTTTA |
| *PPTS* | F | TTCTGTCATGGATCGGACAA |
| *PPTS* | R | TAATAAGATTGGCGGCCTTG |
