# Supplementary material for: Integrated Transcriptomic and Metabolomic Analysis of Five Panax ginseng Cultivars Reveals the Dynamics of Ginsenoside Biosynthesis
Source: Front Plant Sci. 2017 Jun 19;8:1048. doi: 10.3389/fpls.2017.01048 (PMC5474932; doi:10.3389/fpls.2017.01048)
Supplement: Supplementary file 4 [file Table_4.DOCX]

Table S4. Expression levels of genes involved in the MVA pathway in MeJA-treated adventitious roots (CS: Cheongsun, SH: Sunhyang, and SU: Sunun).

| Gene | CS12h | CS24h | SH12h | SH24h | SU12h | SU24h |
| --- | --- | --- | --- | --- | --- | --- |
| *AACT** | 16.14±1.36 | 6.82±0.6 | 5.52±1.71 | 3.08±0.7 | 10.38±4.7 | 13.95±7.44 |
| *MVK* | 1.5±0.29 | 0.84±0.09 | 0.63±0.13 | 0.69±0.05 | 1±0.16 | 0.91±0.17 |
| *HMGR* | 0.98±0.1 | 0.92±0.04 | 0.74±0.07 | 0.84±0.11 | 1±0.08 | 1.35±0.28 |
| *HMGS* | 1.5±0.08 | 0.9±0.12 | 0.61±0.24 | 0.5±0.18 | 0.68±0.28 | 0.69±0.27 |
| *PMK* | 0.58±0.35 | 0.72±0.13 | 0.24±0.04 | 0.42±0.03 | 0.92±0.68 | 0.68±0.28 |
| *MDD* | 1.19±0.4 | 0.81±0.14 | 0.59±0.06 | 0.69±0.1 | 0.97±0.21 | 0.99±0.05 |

Gene expression levels were calculated based on fold changes in expression at 12 and 24 versus 0 h.

*Significant difference (p<0.05).
