# Supplementary figures and images for: Integrated Transcriptomic and Metabolomic Analysis of Five Panax ginseng Cultivars Reveals the Dynamics of Ginsenoside Biosynthesis
Source: Front Plant Sci. 2017 Jun 19;8:1048. doi: 10.3389/fpls.2017.01048 (PMC5474932; doi:10.3389/fpls.2017.01048)

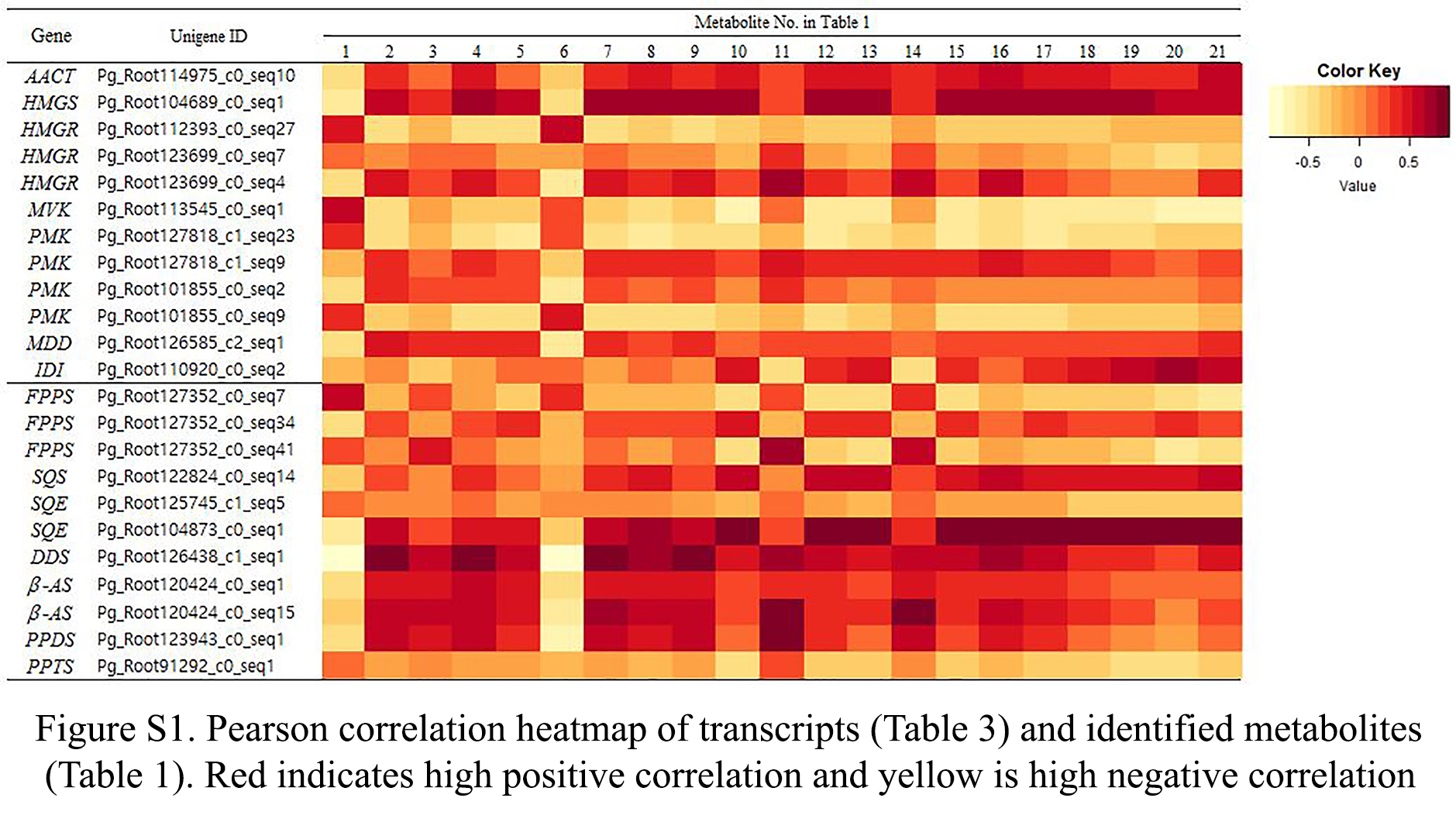

Supplement: Supplementary file 5 [file Image_1.JPEG]
